# Abiotrophia defectiva: A Rare but Critical Cause of Infective Endocarditis

**DOI:** 10.7759/cureus.6492

**Published:** 2019-12-28

**Authors:** Upasana Agrawal, M. Mukhyaprana Prabhu

**Affiliations:** 1 Internal Medicine, Kasturba Medical College, Manipal, IND

**Keywords:** infective endocarditis (ie), abiotrophia, valvular heart disease

## Abstract

Infective endocarditis (IE) refers to an infection involving the endocardial surface of the heart. Most of the cases of IE are known to occur due to infection by viridans Streptococci or Staphylococci species. Abiotrophia defectiva is known to cause less than 1% of cases of IE. Though rare, this organism can cause life-threatening complications such as septic embolization, destruction of heart valves, and heart failure if not detected and treated timely. Here we discuss a case of a 44-year-old female who presented to our tertiary care centre with complaints of fever and easy fatiguability for two weeks. After further investigations, she was finally diagnosed to have IE due to Abiotrophia defectiva. Imaging demonstrated the presence of embolic and valvular complications as well. Through this case, we highlight the importance of early detection and timely management of this condition in order to decrease the occurrence of fatal complications and mortality.

## Introduction

Infective endocarditis (IE) is defined as an infection of a native or prosthetic heart valve, the mural endocardium, or an indwelling cardiac device. Although viridans group Streptococci (VGS) and Staphylococci species are considered as the most common cause of endocarditis, uncommon pathogens may also lead to the disease with significant morbidity and mortality [1]. Though rare, nutritionally variant Streptococci (NVS) are estimated to cause approximately 5%-6% of all cases of IE, including being a major cause of blood culture-negative IE [2]. If not treated early, fatal complications such as heart failure, septic embolization, and valve destruction can occur [1]. Here we present a case of a 44-year-old female who presented with fever and easy fatiguability since two weeks and was eventually diagnosed with IE due to Abiotrophia defectiva. She was found to have developed embolic and valvular complications and was managed medically. The importance of early diagnosis and treatment of this condition has been highlighted in this case report.

## Case presentation

A 44-year-old female with no previous comorbid conditions presented to the emergency department with complaints of fever and easy fatiguability for two weeks and a headache for one day. Fever was high grade, intermittent, not associated with chills and was relieved with medications. It was associated with generalized weakness. There was no history of cough, cold, abdominal pain, loose stools or burning micturition. Her headache had started one day ago and involved the entire left side of the head. No history of vomiting or blurring of vision was present. An episode of altered sensorium had occurred a few days ago where the patient was unable to verbally respond to commands but was able to move her limbs and open her eyes. On admission, the patient was afebrile and had a pulse rate of 70 beats per minute which was regular in rate and rhythm and a blood pressure of 86/50 mm Hg. On systemic examination, no cardiac murmurs were auscultated and no other findings suggestive of IE like clubbed fingers, Janeway lesions or petechiae were found. On central nervous system (CNS) examination, neck rigidity was present and Brudzinski’s sign was found to be positive. Rest of the CNS examination was within normal limits. 

**Figure 1:**
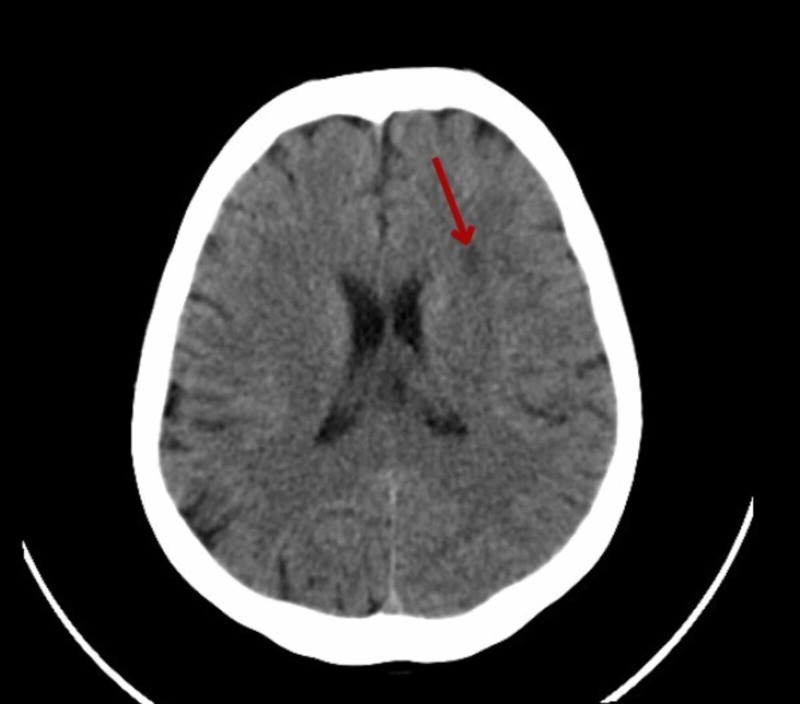
Computed tomography (CT) of the brain showing an ill-defined hypodense area in the deep white matter of the left frontal lobe

**Figure 2:**
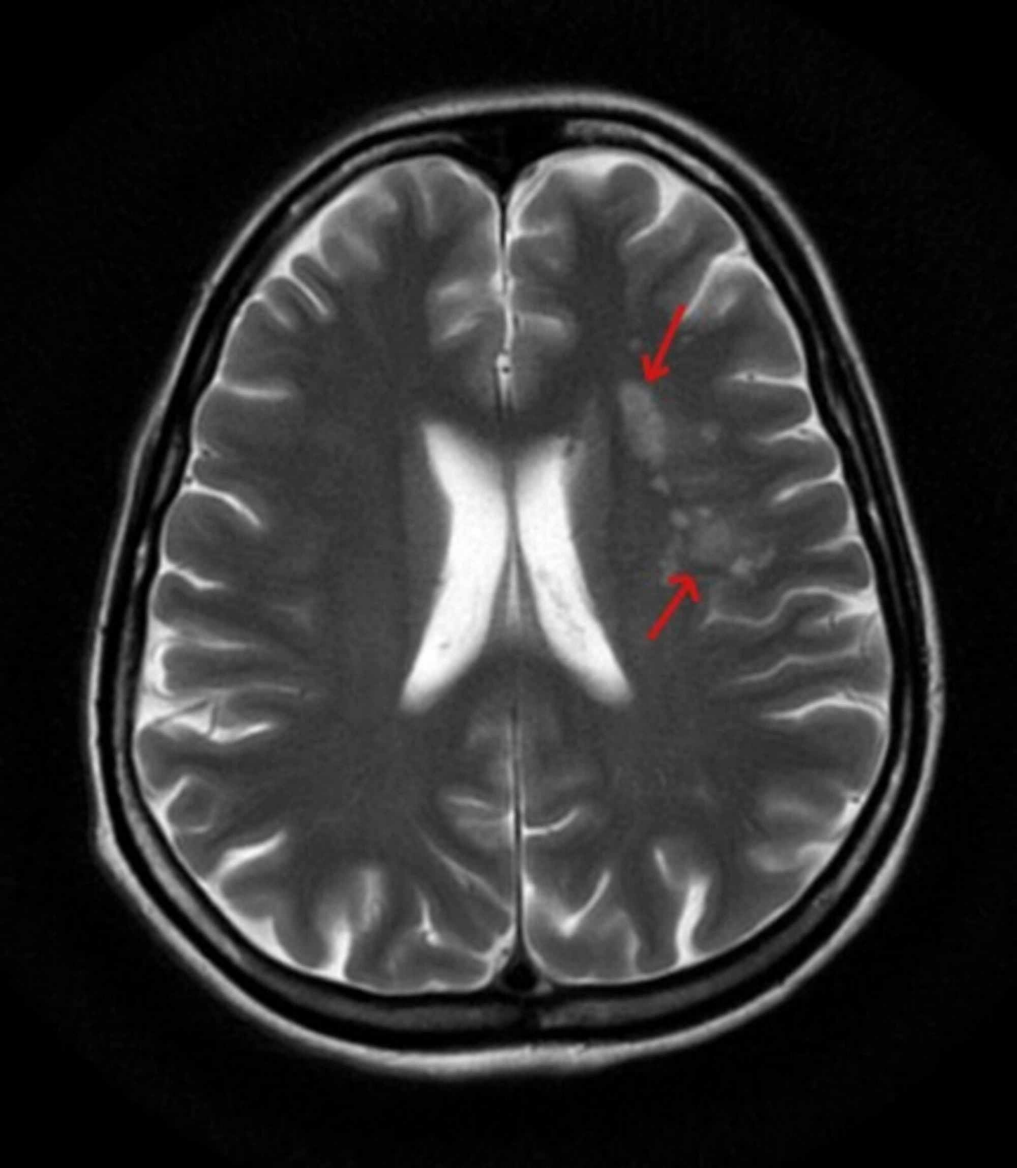
Magnetic resonance imaging (MRI) of the brain showing focal acute infarcts in the left centrum ovale, left corona radiata, and left external capsule in the left middle cerebral artery territory

Laboratory investigations revealed a hemoglobin of 9.9 g/dL, total white blood cell (WBC) count of 11,900/mm^3^ and an erythrocyte sedimentation rate (ESR) of 70 mm/hr. Other viral serology markers were negative. Due to the neck rigidity and Brudzinski’s sign being positive, meningitis was suspected and the patient was started empirically on ceftriaxone, along with other supportive measures. On lumbar puncture, cerebrospinal fluid showed a lymphocytic picture and hence, after neurology consultation, the patient was started on empirical antitubercular therapy (ATT). Ultrasonography (USG) of the abdomen and pelvis showed a wedge-shaped hypo-echoic area seen in interpolar region of spleen suggestive of an acute splenic infarct and an evolving abscess. Mild splenomegaly was also noted. Magnetic resonance imaging (MRI) of the brain showed focal acute infarcts in the left centrum ovale, corona radiata, left external capsule, and left insular cortex in left middle cerebral artery territory. Computed tomography (CT) of the brain showed an ill-defined hypodense area in the deep white matter of the left frontal lobe (Figure 1). A further MRI brain was suggested which showed focal acute infarcts in the left centrum ovale, left corona radiata, and the left external capsule in the left middle cerebral artery region (Figure 2). This lead to the suspicion of IE but her initial blood cultures were sterile. Later her blood culture was found to be positive for Abiotrophic species.

Repeat 2D Echo was done, which showed anterior mitral valve leaflet prolapse and an 8 cm by 4 cm dangling structure, suggestive of vegetation. Moderate to severe mitral regurgitation was noted. Features were suggestive of IE and thus, ATT was stopped. After cardiology consultation, the patient was started on injection ceftriaxone (2 gm, intravenous, twice a day) for six weeks and injection gentamicin (160 mg, intravenous, once daily) for eight days. Due to tachycardia, the patient was started on tablet ivabradine (5 mg, twice daily) for two weeks. At the time of discharge, the patient was stable. She was advised to continue the same intravenous antibiotics and was called for further follow up after two weeks.

In the subsequent outpatient visits, the patient felt symptomatically better. She gained three kilograms of weight. Her ESR decreased from the previous value of 70 mm per hour to 32 mm per hour and her hemoglobin increased from the previous value of 10.7 g/dl to 12 g/dl.

## Discussion

Abiotrophia defectiva, an NVS is an aggressive organism that can cause IE and also has a high probability of causing embolic complications and valvular destruction [3]. Abiotrophia defectiva is a part of the normal flora of the oral cavity, the urogenital and the intestinal tracts [4]. Immunosuppression, pregnancy, and prosthetic valves are the common predisposing factors for this rare infection [5]. In one of the first reported cases of endocarditis due to Abiotrophia defectiva in Korea, the patient who was on post- mitral valve replacement (MVR) had developed the infection following a tooth extraction and was treated successfully with appropriate antibiotics and a second MVR [2]. A case had been reported where a 26-year-old pregnant female was diagnosed to have endocarditis due to Abiotrophia defectiva, secondary to a biofilm-related infection caused by the presence of fixed braces [6]. Our case is unique since the patient did not have any known pre-disposing factors for the disease at the time of presentation. NVS are known to cause 5%−6% of all streptococcal endocarditis cases, out of which <1% of all endocarditis cases are caused by Abiotrophia defectiva [7].

Approximately 125 cases have been published in the literature so far, out of which one published case was complicated by hemophagocytic syndrome [6]. These bacteria do not synthesize pyridoxine, L-cysteine or other essential nutrients required for growth and depend on other bacteria or enriched media to proliferate. Due to this reason, the initial cultures in our study were found to be sterile. Molecular biology technicals allow quicker diagnosis, which can lead to better prognosis [8]. Suspicion regarding this organism should arise in cases of culture-negative endocarditis and additional testing with supplemented media should be performed to encourage growth of colonies [5,9]. A.defective is a pleomorphic organism, appearing as Gram-positive cocci, coccobacilli, and bacilli forms depending on the culture media [10]. Literature suggests that due to the production of a considerable amount exopolysaccharides, the organism has a higher affinity for the endocardium and the ability to bind with fibronectin in the extracellular matrix, further contributes to their virulence [2,9]. 

Infections due to Abiotrophia defectiva are known to cause septic embolization and mortality [11]. Our patient’s case was complicated by the presence of septic emboli in the spleen and also the presence of multiple ischemic foci in the brain. Since these bacteria are also frequently penicillin-resistant, the American Heart Association recommends using the same treatment regime as that used for Enterococcus endocarditis, which consists of ampicillin or penicillin G in addition to gentamicin for a four to six weeks period [12]. Ceftriaxone combined with gentamycin is an alternative option [11]. This combination, involving intravenous ceftriaxone and gentamicin was successfully used to treat our patient. 

Despite the sensitivity to antimicrobials, approximately 50% of patients need surgical management [13]. Lin et al. published a study involving eight cases of NVS endocarditis, out of which seven of the cases were associated with large vegetation size, that is, greater than 10 mm. Eventually, seven of the eight cases went on to have valve replacement, performed either because of vegetation size or severe heart failure [14]. A rare case of a quadruple valve IE due to Abiotrophia defectiva has also been reported in a patient with pre-existing ventricular septal defect. The patient was successfully managed surgically [9]. In our case report, the patient was managed medically with appropriate antibiotic therapy and has been feeling symptomatically better during the follow-up visits. 

## Conclusions

Even though Abiotrophia defectiva is a rare cause of IE, it causes higher mortality rates as compared to other organisms like viridans Streptococci. Most reported deaths due to Abiotrophia related endocarditis are due to valvular disease, acute heart failure or major systemic embolization. Hence, early suspicion, diagnosis and aggressive treatment of this culture-negative endocarditis is important to prevent the fatal complications and have a better prognosis for the patients.
